# Two-Stage Nerve Graft Using a Silicone Tube

**DOI:** 10.3389/fsurg.2015.00012

**Published:** 2015-04-23

**Authors:** Shayan Abdollah Zadegan, Masoumeh Firouzi, Mohammad Hossein Nabian, Leila Oryadi Zanjani, Reza Shahryar Kamrani

**Affiliations:** ^1^Tissue Repair Laboratory, Institute of Biochemistry and Biophysics (IBB), University of Tehran, Tehran, Iran; ^2^Research Center for Neural Repair (RCNR), University of Tehran, Tehran, Iran; ^3^Department of Orthopedic and Trauma Surgery, Shariati Hospital, Tehran University of Medical Sciences, Tehran, Iran; ^4^Joint Reconstruction Research Center, Tehran University of Medical Sciences, Tehran, Iran

**Keywords:** peripheral nerve injuries, tendon injuries, autografts, fibrosis, nerve regeneration

## Background and Significance of the Problem

Peripheral nerve injury (PNI) is a life altering situation with significant morbidity (1). Majority of PNI cases occur in the upper limb (2, 3), where the sensory/motor dysfunction and pain, leads to psychological distress and substantial disability in daily socio-economic activities (1, 4–6).

In patients with severe concomitant soft tissue injuries, the primary repair of the nerve gap with a graft is usually impossible or unsuccessful (7, 8), mainly due to extent of the injury, distortion of the normal topography, excessive scarring, and subsequent adhesion and tethering (7).

A similar situation is also present in reconstruction of scarred flexor tendon system of the hand. Adhesion of the injured tendon to the surrounding soft tissues is a challenge for the hand surgeons. A widely accepted treatment for this condition is two-stage tendon graft (9). This technique was introduced previously to improve the outcome of the tendon reconstruction in unfavorable conditions (10). In 1965, Paneva-Holevich reported a “two-stage tenoplasty,” which was modified by Hunter and Salisbury in 1971 (11, 12). The modified technique involved the use of a silicone rod implant in the first stage and grafting through the pseudosheath formed around the silicone in the second stage with a 3–6 month interval (9). This technique of flexor tendon repair was used in different clinical conditions in which the primary repair was not possible or failed. The results were satisfactory and the approach was proved to be reliable for restoring flexor tendon function (9). With the silicone tube implantation in the first stage, a sheath would form around the silicone due to its non-absorbable nature (13). After removing the silicone tube in the second stage, the severely scarred tendon bed would transform into a “smooth well-organized pseudosheath through which a tendon can glide” (14). In this article, we would like to highlight the potential benefits of using a similar two-stage technique in nerve grafts of severely traumatized limbs.

## Current State of the Art and its Limitations

Although nerve repair strategies have been developed in the last decades, the outcome is still suboptimal and unpredictable, especially in unfavorable conditions (15, 16). In the neurotmesis, primary end-to-end repair is the most commonly used technique and the gold standard surgical treatment (17). In presence of a large defect, two nerve ends cannot be approximated without tension. Consequently using a graft or conduit as a bridge will be essential under such circumstances (7). Despite the disadvantages of autograft, such as donor site morbidity and limited length of available graft material, this approach is still the gold standard for nerve bridging; especially for long gaps (>3 cm) (7, 8, 17–19). Shorter gaps can be repaired with different types of conduits. This eliminates the disadvantage of donor site morbidity (7). Various biologic materials such as bone, artery, vein, and skeletal muscle are used for conduits as well as synthetic materials (7, 18). Synthetic materials with absorbable nature (e.g., polyglycolic acid polymer) are preferred over non-degradable ones (e.g., silicone); because in long term, chronic foreign body reaction produces excessive scar which can interfere with the nerve function and cause the need for the secondary surgical intervention (13, 20).

In severe injuries or infected wounds, nerve defect repair is more challenging (7, 8). In such cases, after the primary marking of the proximal and distal ends of the nerve, a delayed nerve grafting (approximately 3 weeks to 3 months post-injury) is performed in a secondary procedure (8).

## Proposed Strategy and Advantages Over the Current Nerve Repair Approaches

We propose a two-stage graft approach in repair of the peripheral nerve gaps in severely traumatized limb:

- In the first stage (the primary phase of injury/initial exploration), proximal and distal ends of the transected nerve are determined and the nerve gap is temporarily reconstructed with interposition of a silicone tube.
- In the second stage (after 1–3 months), the silicone tube undergoes encapsulation with the pseudosheath. In this stage, the silicone tube is detached from the surrounding membrane and pulled out without causing any damage to the membrane. The nerve graft segment is interposed through the pseudosheath and the nerve ends are sutured to the ultimate nerve graft.

It is evident from previous studies that the pseudosheath formed around the silicone is not simply a scar layer. Hunter and colleagues showed in an experimental study in a primate model that after 8 weeks, the pseudosheath consists of three layers: (1) the intima that was formed with secretory cells containing vacuoles of glycosaminoglycan that provided a soft and sliding surface, (2) the media that was rich in collagen and provided vascular support, and (3) the adventitia that was highly vascular with loose fibrous tissue (21). The authors described the pseudosheath as a “morphologically stable structure that showed no propensity for longitudinal contracture” (21). The evidence supports the idea that this vascular sheath could provide a suitable bed for the interposed nerve segment (22), as it did for the tendon graft.

In 1979, Lundborg and colleagues evaluated nerve regeneration through a pseudosheath conduit (23). A silastic rod wrapped with a stainless steel spiral was implanted subcutaneously in the back of the rats. After 3 weeks, the produced pseudosheath was transferred and used as a conduit to bridge a 10–12 mm length gap in the sciatic nerve. Assessment of regeneration after 3 months showed a nerve trunk with small fascicles, surrounded by an epineurium, without any wild uncontrolled growth of axons. In 1980 and 1981, they evaluated the same procedure with electromyography and reported a good functional regeneration of motor fibers (24, 25). In 1985, Mackinnon and colleagues used the staged procedure described by Lundborg for ulnar nerve regeneration in a primate model (26). After 6 weeks interval, a coiled wire ensheathed by pseudosheath was produced. A 3-cm segment of the ulnar nerve was excised and the distal and proximal end of the nerve were inserted into the sheath and sutured. After over 9 months of observation, they found regeneration of the primate nerve across a 3-cm gap through the pseudosheath. However, no functional assessment was done and the regeneration across the gap after 9 months was of poorer histologic quality compared with the grafting techniques of that time (26). In 1988, in a more comprehensive study, they found that the quality of regeneration between Sural nerve graft and the pseudosheath was similar (27). Although the basic idea of using pseudosheath as a conduit was brilliant, it lost its importance after new conduits were introduced.

## Objective Summary of the Promise and Pitfalls of the Two-Stage Technique

In two-stage nerve graft, the pseudosheath formed around the silicone tube during the first stage is used as a tunnel to envelope the nerve graft segment in the second stage. This pseudosheath is a suitable bed for the interposed nerve graft because of: (1) the soft and sliding lumen that prevents adhesion and tethering of the interposed nerve graft, (2) good vascularization that provides oxygen and nourishment for the graft, (3) the stable structure that protects the interposed nerve graft from compression.

The most important parameter in this technique is the time between the two stages. This factor plays an influential role in both the nerve regeneration and the pseudosheath maturation. Previous studies showed that axonal regeneration decreases with increasing time after the nerve trauma; mainly due to the decreasing capacity of proximal axons to extend as well as lower proliferation rate and neurotrophic factor production of distal Schwann cells (28). The optimal timing for regeneration is different between animal and human studies. In animal models, axonal regeneration begins to reduce between 1 and 2 months post-injury (29–32) while in clinical studies this time range is 3–5 months (7, 33, 34). This optimal timing of nerve regeneration is synchronous with the proper time needed for maturation of the pseudosheath. In clinical practice, the minimum time interval between two stages of tendon graft is 2–3 months to permit structural maturation of the pseudosheath (35, 36). However, electron microscopy in an experimental study on hen shows that after 4 weeks, a pseudosheath comparable to normal parietal sheath is formed around the silicone tube (37).

Clearly, there are two major drawbacks to this technique. The first drawback is the delay of one to three months for grafting the nerve, due to the need for a two-stage operation. The other drawback to this technique is the possible damage to the formed pseudosheath in the second stage. Normally, this technique is not an alternative to nerve grafting when it is possible but it is a solution when the primary nerve grafting is not possible or is disappointing, because of the surrounding scar tissue, which makes it illogical to do primary nerve graft. We believe that the pseudosheath can be beneficial when the nerve defect is large and the outcome of traditional nerve graft or conduits is expected to be disappointing.

In conclusion, this technique combines the advantages of the old technique of the nerve graft and the new technique of the nerve conduit without losing the potential advantages of neither of them. It is expected that two-stage nerve graft technique will reduce the adverse effects of the scar in acute phase and improve the outcome of grafting in unfavorable conditions. Future experimental and clinical studies are needed to validate and expand this proposed technique.

## Conflict of Interest Statement

The authors declare that the research was conducted in the absence of any commercial or financial relationships that could be construed as a potential conflict of interest.
